# Space Charge Characteristics of Polypropylene Modified by Rare Earth Nucleating Agent for *β* Crystallization

**DOI:** 10.3390/ma12010042

**Published:** 2018-12-24

**Authors:** Jiaming Yang, Mingze Gao, Hong Zhao, Shilin Liu, Ming Hu, Shuhong Xie

**Affiliations:** 1 Key Laboratory of Engineering Dielectric and its Application, Ministry of Education, Harbin University of Science and Technology, Harbin 150080, China; gaomingze00@163.com (M.G.); shilinliu11@163.com (S.L.); 2 Zhongtian Technology Submarine Cable Co., Ltd., Nantong 226000, China; hum@chinaztt.com (M.H.); xiesh@chinaztt.com (S.X.)

**Keywords:** rare earth nucleating agent, polypropylene, crystalline morphology, space charge, charge trap

## Abstract

Compared to cross-linked polyethylene, polypropylene has a thermoplastic property and the advantage of recycling. However, the poor impact resistance at low temperature and the corresponding space charge problem restrict the application of polypropylene with the extruded high voltage direct current (HVDC) cable. Sufficient introduction of the *β* form of the polypropylene crystal can significantly improve impact resistance at low temperatures. Although it has been widely applied in insulation engineering, the effect of *β*-crystal on the space charge characteristics of polypropylene has rarely been researched until now. In this paper, a rare earth nucleating agent of *β*-crystal is employed to modify the performance of polypropylene to investigate the effects of nucleating agent content on *β*-crystalline, mechanical relaxation, trap, and space charge characteristics of polypropylene. The results of differential scanning calorimeter (DSC) and X-ray diffraction (XRD) tests indicate that the relative content of *β*-crystal in modified polypropylene increases gradually with the increasing concentration of the nucleating agent, approaching 43.5% when the nucleating agent content has been raised to 0.2 wt %, suggesting appreciable efficiency of the nucleating agent utilized in our research. Scanning electron microscopy (SEM) is utilized to characterize the morphology of *β*-crystal spherulites, which illustrates that the *β*-spherulites are in bunchy shape, and the lamellar crystals are parallel to each other without an obvious boundary between them. The results of the space charge test demonstrate that the modified polypropylene can substantially suppress space charge accumulation, which is attributed to an increment of *β*-crystal content by adopting a rare earth nucleating agent. It is indicated from dynamic mechanical analysis (DMA) measurements that the enhancement of *β*-crystalline in modified polypropylene can distinctly increase and decrease the *β* and *α* relaxation losses, respectively, which proves that the defects in *β*-crystal and amorphous regions are reduced and increased respectively. Thermally stimulated depolarization current tests further confirm that the number of traps caused by defects in the *β*-form of polypropylene crystal declines definitely, which dominantly accounts for the suppression of space charge accumulation.

## 1. Introduction

In recent years, with the increase of energy demand, recycling cable insulation materials have attracted tremendous attention [1,2]. Unlike cross-linked polyethylene, which has been widely used in power cables, polypropylene (PP) is a typical thermoplastic material and can be recycled after melting at high temperature. However, the poor impact strength at low temperatures of polypropylene limits its applications in extruded high-voltage power cables [3,4]. As a semi-crystalline polymer, PP can crystallize to five crystal structures under different crystallization conditions, among which the *α*-crystal and *β*-crystal are the two most common crystal forms [5,6,7,8,9]. Pure PP usually crystallizes in the *α*-crystal, while in comparison the *β*-crystalline PP represents excellent ductility and obviously improved impact strength [10,11,12]. Adding a nucleating agent of *β*-crystalline (*β*-nucleating agent) is a feasible way to obtain high *β*-crystal content in PP with high stability and reliability [13,14,15].

Space charges can easily accumulate in the polymer insulating materials, restricting the actual operation in high voltage direct current (HVDC) cables [16,17,18,19]. The crystallization morphology of PP is easier to control than that of polyethylene, and the crystallization temperature and cooling rate will affect the crystallization behavior of PP [20,21]. It has been shown that the spherulite size in PP significantly affects the electric performance [22,23]. Studies have shown that when spherulites grow, impurities, non-crystallizable, or poorly crystallizable materials are pushed ahead of the growing spherulitic front and collect at the spherulitic boundaries, becoming weak points and causing changes in mechanical and electrical properties [24,25,26]. By controlling the thermal history of the crystallization process, the morphology of polyolefin changed, and the electrical properties were improved [27,28]. The electrical properties of semi-crystalline polymer materials are highly dependent on evolutions in crystalline morphology. By adding different kinds of nucleating agents to PP, the crystallization behavior and morphology can be modified as desired to improve the electrical properties. At present, researchers rarely pay attention to the electrical properties of *β*-crystalline PP. Wu modified the PP using an amide *β*-nucleating agent and found that the space charge of the modified material decreased, and the mechanism may be related to the variation of the trap parameters and morphology of the modified material [29]. However, the reason for the change of trap parameters and its relationship with crystalline morphology remains unclear. Whether different kinds of nucleating agents such as rare earth nucleating agents can also improve the space charge suppression ability of PP remains to be studied. In this paper, a rare earth *β*-nucleating agent nominated by WBG-II is employed as the modifier of polypropylene. The effects of the amount of nucleating agent on the content of *β*-crystal, mechanical relaxation, and space charge characteristics of polypropylene were investigated. The trap distribution in *β*-crystalline PP is analyzed by means of a thermally stimulated current method to elucidate the mechanism of suppressing space charge accumulation in *β*-crystalline PP.

## 2. Materials and Methods

### 2.1. Material Preparation

The homopolymer PP of model T30S produced by Sinopec (Daqing, China) is used as a matrix material with the rare earth *β* nucleating agent of WBG-II produced by Guangdong Wei Linna functional material Co., Ltd. (Foshan, China), and the antioxidant of Irganox 1010 produced by BASF Gao-Qiao Performance Chemicals (Shanghai, China). The individual 0.05%, 0.2%, and 0.5% contents of WBG-IIand the 0.3% content of the antioxidant are blended with T30S under 190 °C. Specific mixing methods are as follows: After the temperature of mixer rises to 190 °C, the PP material is firstly put in and 3% antioxidant is added after two minutes, and nucleating agent after one minute of mixing. After mixing processing for 8 min, the material prepared by mixing is compacted under 15 MPa pressure by flat plate press at 190 °C.

### 2.2. Characterization and Testing Scheme

Morphology studies of the PP and *β*-PP samples with the thickness of 100 μm were placed between two microscope slides and were then observed with an optical microscope (Changfang XPV-400E, Shanghai, China) in polarization mode. 

The microstructures of the prepared samples are characterized using a Hitachi SU8020 Scanning electron microscopy (SEM, Tokyo, Japan). The flat plate samples with thickness of 1mm were fractured in liquid nitrogen, and then their cross sections were etched with the mixture of 50 mL concentrated sulfuric acid, 20 mL concentrated phosphoric acid, 5mL water, and 0.75 g potassium permanganate for 4 h, and then washed using the cleaning solution consisting of concentrated sulfuric acid, water, and hydrogen peroxide in volume ratio of 2:7:1. After this, all samples were sputter-coated with platinum to avoid the accumulation of charge. Finally, the cross-section of the treated sample was observed by SEM.

The crystalline characteristics of PP and its blends were tested by a differential scanning calorimeter of DSC822e produced by Mettler Toledo, Zurich, Switzerland. In the experiment, after loading 5 mg of the sample, the aluminum crucible was heated from ambient temperature to 190 °C at the speed of 10 °C/min and kept for 2 min, then cooled down to 30 °C at the rate of 10 °C/min in which the crystallization curve was measured. After maintaining a constant temperature for 2 min, the melting curve was obtained by heating up to 190 °C at a speed of 10 °C/min.

The X-ray diffraction spectra in the 10–30° scanning range for the sample with a thickness of 0.1 mm was obtained by exploiting the XRD scanner of the EMPYREANE produced by the PANaltical company (Amsterdam, The Netherlands) to analyze the crystal phase comprehensively.

Dynamic mechanical properties were measured using a dynamic thermomechanical analyzer of DMAQ800 manufactured by TA instruments (New Castle, DE, USA). The tensile mode and the target amplitude of 10 μm are adopted in measurement for the samples with the thickness, length, and width of 1 mm, 15 mm, and 8 mm, respectively. The samples were cooled to −80 °C for 2 min and then temperature increased linearly from −80 °C to 160 °C at the heating rate of 3 °C/min in which the storage modulus (*E*’), loss modulus (*E*’’) and loss factor (tan δ) were tested.

Space charge distribution was measured using the pulsed electro-acoustic method (PEA) as implemented with the testing system produced by Shang Hai Xiangtie electromechanical device Co., Ltd., Shanghai, China. The spatial resolution of this PEA system is about 18 μm. Aluminum electrodes were deposited onto both surfaces of the specimens by sputtering. The diameter of an aluminum electrode was 25 mm and the thickness of specimens was 0.3 mm. The space charge distribution of the PP was measured under 40 kV/mm and a short-circuit for 30 min with direct current applied voltage, and all measurements were performed at room temperature (25 ± 3 °C). In order to qualify for the repeatability of experimental results, all tests were performed three times under the same conditions.

The charge trap characteristic was tested using the thermally stimulated depolarization current (TSDC) method. Aluminum electrodes were deposited onto both surfaces of the specimens by sputtering. The diameter of the aluminum electrodes was 25 mm, and the thickness of the specimens was 0.1 mm. The prepared samples were heated to 60 °C in a vacuum environment and then were electrically polarized by applying 40 kV/mm DC high voltage at this temperature for 30 min. The liquid nitrogen was then used for rapid cooling the samples to below −50 °C so that all kinds of charge carriers had been "frozen", after which the DC high voltage was removed and also the short-circuit the sample for about 10min. After that, the sample was heated linearly under a constant heating rate of 3 °C/min and the short-circuit current was measured using a 6517B electrometer (Keithley Instrument Inc., Cleveland, OH, USA). 

## 3. Results and Discussion

### 3.1. Crystalline Features

**Figure 1:**
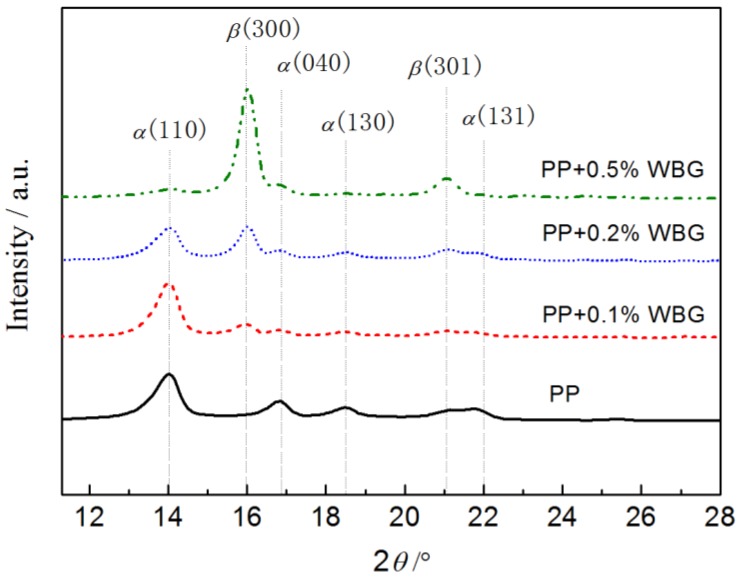
X-ray diffraction spectra of polypropylene (PP) and *β*-PP.

The X-ray diffraction spectra of the *β*-crystalline PP (*β*-PP) samples prepared with different amounts of *β*-nucleating agent are shown in Figure 1. It is noted that two characteristic diffraction peaks of the *β*-crystal at 16.0° and 21.1° significantly increase in intensity with the increasing content of the *β*-nucleating agent, while the peak values of the *α*-crystal diffraction peaks at 14.0°, 18.5°, and 21.8° decrease gradually. MDI Jade 6.5 (Materials Data, Inc., Livermore, CA, USA) was used to subtract the amorphous background and to fit the crystalline peaks using the pseudo-Voigt profile. According to the intensity of each fitted diffraction peak, the relative content of *β*-crystal K*_β_* in PP can be calculated with the Turner-Jones formula [30]:

(1) $$K_{\beta }=\frac{H_{\beta \left(300\right)}}{H_{\alpha \left(110\right)}+H_{\alpha \left(040\right)}+H_{\alpha \left(130\right)}+H_{\beta \left(300\right)}}\times 100\%$$

where *H_α_*_(110)_, *H_α_*_(040)_, and *H_α_*_(130)_ denote the intensities of *α*-crystal diffraction peaks from the (110), (040), and (130) crystallographic planes, respectively, and *H_β_*_(300)_ represents the intensity of the *β*-crystal diffraction peak from the (130) crystallographic plane, and *K_β_* symbolizes the relative content of the *β*-crystal. It is explicitly indicated from the XRD analysis results of the pure PP and *β*-PP as listed in Table 1 that the relative content of the *β*-crystal approaches to 43.5% when the amount of nucleating agent is raised to 0.2 wt %, verifying the high efficiency of the rare earth nucleating agent used in our work.

**Figure 2:**
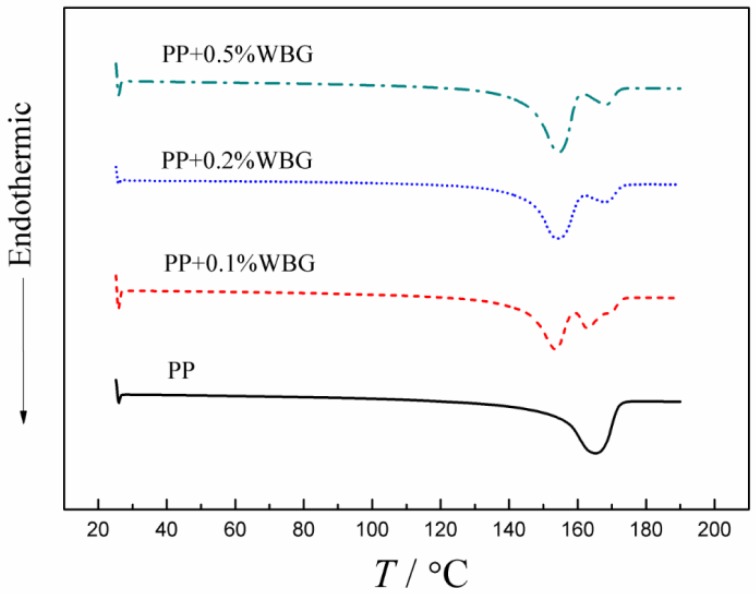
Differential scanning calorimeter (DSC) traces of the melting behavior of iPP and *β*-PP.

The tested differential scanning calorimeter (DSC) traces of the 2nd melting behaviors of iPP and *β*-PP with different contents of WBG are illustrated in Figure 2, which implies that the pure PP has only one melting peak of the *α*-crystal at about 165 °C, while the area of the *α*-crystal melting peak of *β*-PP decreased obviously after exploiting the WBG nucleating agent. Moreover, a larger area melting peak appears at about 155 °C, characterizing the *β*-crystal in *β*-PP, the center temperature (identify melting point) of which slightly rises with the increment of the nucleating agent content. When the nucleating agent is added in low amounts, PP molecules crystallize in the *β*-crystal without sufficient crystalline perfection, and the size of the spherulite is relatively small. Whereas, with the increase of the nucleating agent concentration that signifies achievement of a higher density of crystal centers, the growth of the *β*-crystal is accelerated and promoted in the crystalline order, consequently resulting in the rise of the melting point.

### 3.2. Crystalline Morphology

**Figure 3:**
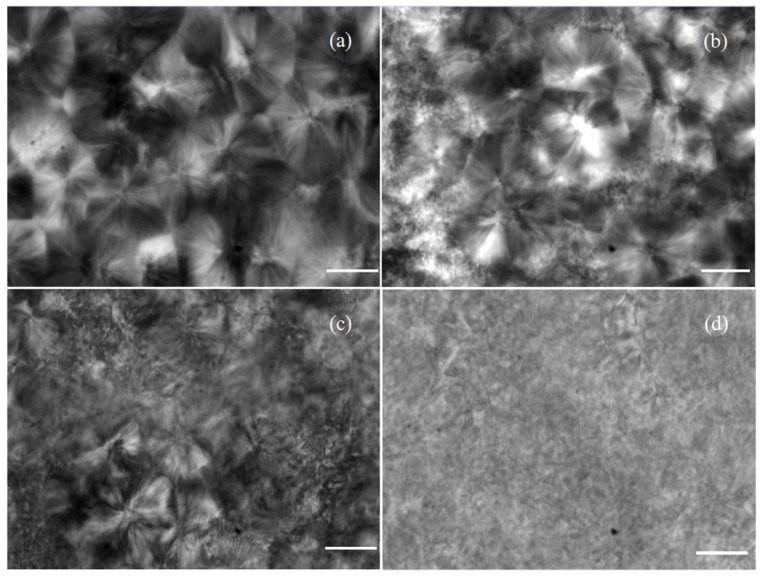
Polarizing microscopy images of PP and *β*-PP (scale bar = 20 μm): (**a**) Pure PP; (**b**) 0.1% WBG; (**c**) 0.2% WBG; (**d**) 0.5% WBG.

**Figure 4:**
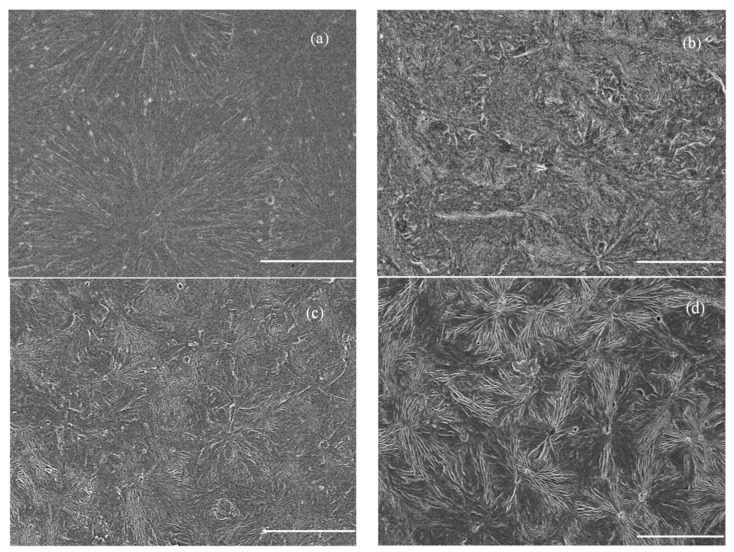
SEM images of PP and *β*-PP (scale bar = 20 μm): (**a**) Pure PP; (**b**) 0.1% WBG; (**c**) 0.2% WBG; (**d**) 0.5%WBG.

The results of polarizing microscope analysis for pure PP and *β*-PP are shown in Figure 3. The pure PP dominantly crystallize in the form of *α*-crystals with a clear grain boundary between the spherulites, while the amounts of *β*-crystal and *α*-crystal of *β*-PP increases and decreases respectively and the boundary between spherulites becomes blurred with the increase in the addition of *β*-nucleating agent WBG. After etching treatment, the surfaces of the pure PP and *β*-PP are characterized using SEM as shown in Figure 4. It is illustrated that the lamellae grow from the center along the radial direction with an obvious boundary for *α*-spherulites. With the content increase of the WBG nucleating agent, the regularity of *α*-spherulites decreases considerably, while the density of *β*-spherulites in bundle shape evidently increases, in which the lamellae of *β*-spherulites are parallel to each other without an obvious boundary between the spherulites. Furthermore, when the content of *β*-crystal is only 0.1%, the sizes of lamellae and spherulites of *β*-crystal are apparently smaller with discrepant morphology, which means that the crystallization process of *β*-crystal is not perfect. With the increase of the nucleating agent content, it can be observed that the sizes of *β*-crystal spherulites and their lamellae increase. When the nucleating agent content reaches 0.5%, the dimensions of *β*-crystal are uniform and regular in shape. The results of crystal morphology characterization are consistent with the testing results of XRD and DSC methods.

### 3.3. Space Charge Characteristics 

**Figure 5:**
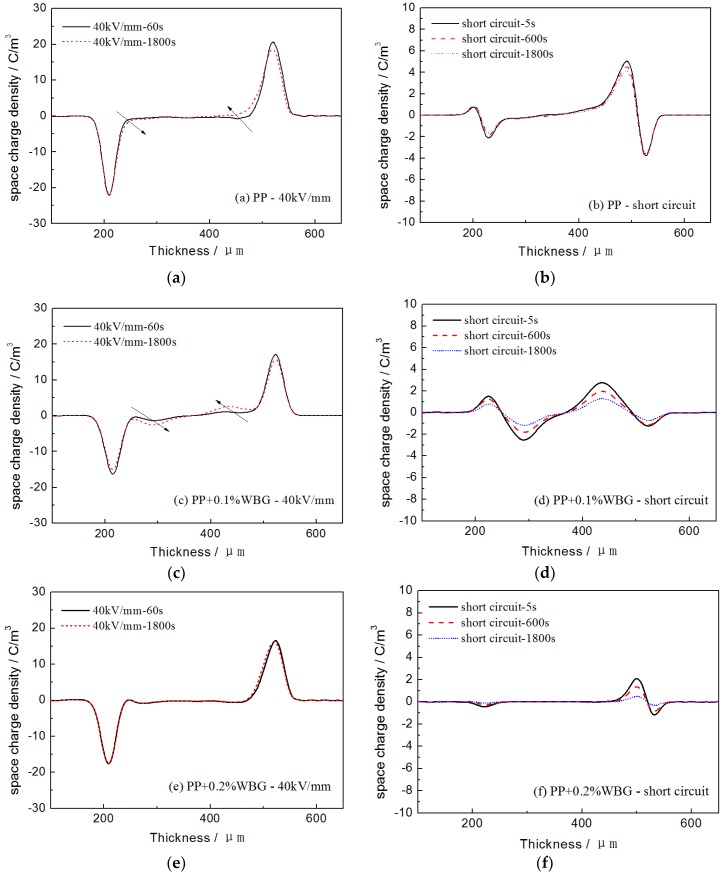

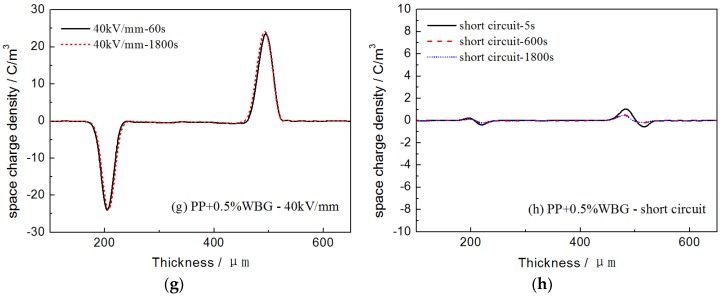
Space charge distributions of PP and *β*-PP under the 40 kV/mm electric field (left panels: (**a**) Pure PP; (**c**) PP + 0.1% WBG; (**e**) PP + 0.2% WBG; (**g**) PP + 0.5% WBG) and short circuit conditions (right panels: (**b**) Pure PP; (**d**) PP + 0.1% WBG; (**f**) PP + 0.2% WBG; (**h**) PP + 0.5% WBG).

The space charge distributions of pure PP and *β*-PP are tested with the results of the characteristic curves shown in Figure 5. It is noted from Figure 5a that charges are rapidly injected near both the cathode and anode in pure PP with the increase of polarization time under the 40 kV/mm field. In comparison, the space charge distribution under the short circuit, as indicated in Figure 5b, clearly represents the space charge quantity and its isothermal decay with time, showing that the peak densities of positive and negative space charges are 5 C/m^3^ and −2 C/m^3^, respectively, with low decay rates. After 1800 s time of short circuit treatment, the density of positive space charges retains with about 4 C/m^3^, demonstrating that the traps in the materials are deep, and thus the space charges decayed slowly. After the addition of the 0.1% WBG nucleating agent, as the space charge distributions of *β*-PP under the 40 kV/mm field and short-circuit conditions respectively show in Figure 5c,d, the number of space charges is reduced while the depth of charge injection is increased in comparison with pure PP. Both the positive and negative space charges gradually migrate inward to 0.1% WBG *β*-PP with the increasing applied voltage time, as exhibited in Figure 5c. The decaying rates of the space charges in *β*-PP are higher than those of pure PP, as illustrated in Figure 5d for the short-circuit. These experimental results prove that the trap level in *β*-PP decreased compared with pure PP, leading to increased charge mobility. With the addition of the 0.2% WBG nucleating agent, as the space charge distributions of *β*-PP under the applied field and short-circuit are respectively shown in Figure 5e,f, the number of space charges decreases further, with the peak positive space charge density declining to about 2 C/m^3^, and the space charges decay faster than those of the pure PP, almost disappearing inside *β*-PP after 1800 s under short circuit conditions. As the nucleating agent content reaches 0.5% with the results shown in Figure 5g,h, space charges can hardly be injected into the material with only 1 C/m^3^ of the maximum density, and they decay rapidly to 0 after 1800 s under short circuit conditions. 

**Figure 6:**
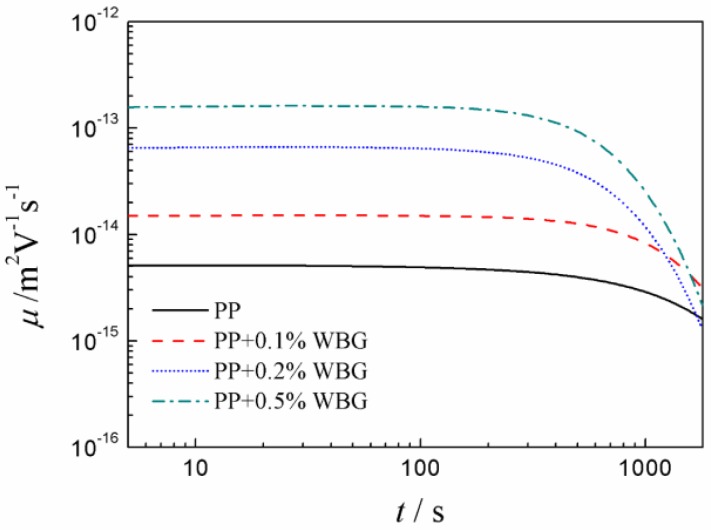
The apparent mobility values of PP and *β*-PP.

**Figure 7:**
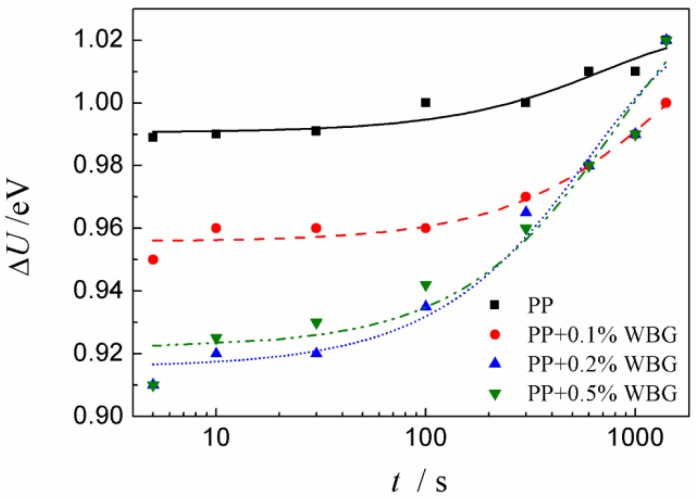
Values of trap depth of PP and *β*-PP.

Based on the method provided in Reference [31], the apparent carrier mobility and trap depths of PP and *β*-PP were calculated from the space charge of the isothermal decay data, and the results are shown in Figure 6 and Figure 7. It can be seen from Figure 6 that as the WBG content increases, the mobility of *β*-PP increases significantly. As the short circuit time increases, the mobility of *β*-PP gradually decreases and approaches the mobility of the PP. As shown in Figure 7, the apparent trap depth of *β*-PP is lower than that of PP. As the short circuit time increases, the charge in the shallow traps gradually releases, and the remaining space charge is mostly captured by the deep traps. As can be seen from the above discussion, the mobility of *β*-PP is increased, and the trap level becomes shallower than that of the PP. It is shown in Figure 4 that the morphology of *β*-crystal is relatively loose, and the melting trace of *β*-PP shown in Figure 2 further indicates that the melting temperature of the *β*-crystal is low, meaning the order and regularity of the *β*-crystal are not as good as *α*-crystal. Since the size of the *β*-crystal is small, more crystal boundaries are formed, which causes the charge to migrate more easily in the *β*-crystal and crystal boundaries of *β*-PP. 

### 3.4. Mechanical Relaxation Properties

**Figure 8:**
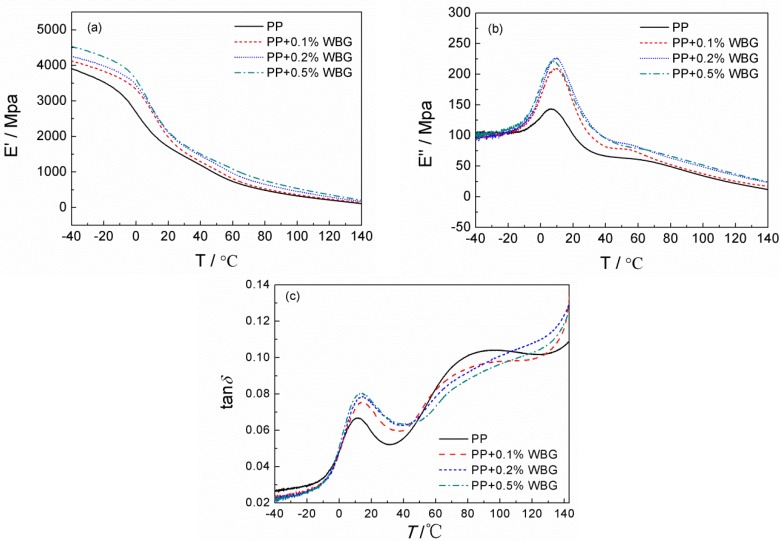
Dynamic mechanical analysis (DMA) spectra of PP and *β*-PP: (**a**) Storage modulus; (**b**) loss modulus; (**c**) loss factor.

The storage modulus, loss modulus, and loss factor of PP and *β*-PP are shown in Figure 8. It can be seen from Figure 8a that the storage moduli of the PP and *β*-PP are relatively close in the high-temperature range, while the storage modulus of *β*-PP is higher than that of the pure PP in the low-temperature range, increasing with the nucleating agent content. The crystal morphology of Figure 4 shows that the structure of the *β* crystal is not as dense as that of the α crystal, and the inter-lamellar gap of the *β* crystal is likely to exhibit more amorphous phases than PP. At lower temperatures, the amorphous phase in the inter-lamellar is surrounded by the crystalline phase, so it does not significantly reduce the storage modulus, making the storage modulus of *β*-PP higher than that of PP. As the temperature increases, the interaction between lamellas weakens, and the amorphous phase between the lamellas begins to cause a decrease in the elastic modulus. As can be seen from Figure 8b of the loss modulus, as the nucleating agent content increases, the loss modulus increases. The relaxation loss spectra of pure PP and *β*-PP are measured with the results shown in Figure 8c. The two loss peaks at about 15 °C and 85 °C originate from the *β* and *α* relaxation processes essentially attributed to the glass transition and the chain relaxation in the crystal regions, respectively [32]. As compared with the peak amplitude and locations of *β* loss existing in pure PP and *β*-PP, the *β* relaxation loss of *β*-PP is higher than that of pure PP. However, the varying trend of the *α*-relaxation loss peak is opposite to that of the *β*-relaxation loss for the two materials. Both the intensity and temperature position of the *α*-relaxation loss peak of *β*-PP are lower than for pure PP, which decreases with the increase of the *β* nucleating agent content, as shown in Figure 8c.

It is concluded from the above analysis that the nucleating agent can promote the crystallization process of *β*-crystal in the PP. The structural defects in the *β*-PP crystal region are remarkably impeded by sufficient crystallization so that the *α*-relaxation loss is lower than that of the pure PP. Meanwhile, the dimension of the *β*-spherulites is distinctly smaller than that of the *α*-spherulites. Therefore, after the nucleating agent is utilized, the larger *α*-spherulites are substituted by small-scale *β*-spherulites, resulting in a higher density of the crystal/amorphous interface due to the higher surface/volume ratio for the smaller *β*-spherulites. Sine the transition zone from the crystalline to the amorphous state is more likely to cause the formation of structural defects, the *β* relaxation loss of *β*-PP is notably increased.

From the images of the polarized light microscopy and scanning electron microscopy shown in Figure 3 and Figure 4, it is implied from the ambiguous grain boundary that the binding of the crystal region to the amorphous region in *β*-PP is slightly stronger than that of the pure PP, giving rise to a slight increment of *β*-relaxation loss temperature. It is also found from Figure 3 and Figure 4 that the structure of *β*-spherulites is relatively loose, and thus the peak position of *α*-relaxation loss occurs at a lower temperature than that of the pure PP. The DMA results demonstrate that WBG can change the distribution characteristics of defects in crystal and amorphous regions by inducing *β*-crystal into PP, and the defect densities in the crystal and amorphous regions have been decreased and increased respectively.

### 3.5. Charge Trap Characteristics

Thermally stimulated currents can be used to analyze trap features in materials with the principle of applying a polarization voltage on the material to fully establish space charge polarization, after which the temperature of the tested sample is rapidly reduced to a very low temperature by using liquid nitrogen to “freeze” the trapped charges, and then increased at a constant heating rate to gradually thaw the trapped charges. This forms the current recorded as a function of temperature (TSDC spectrum) [33,34]. Based on the method provided in Reference [35], trap depth and density distribution are approximated from the TSDC data.

**Figure 9:**
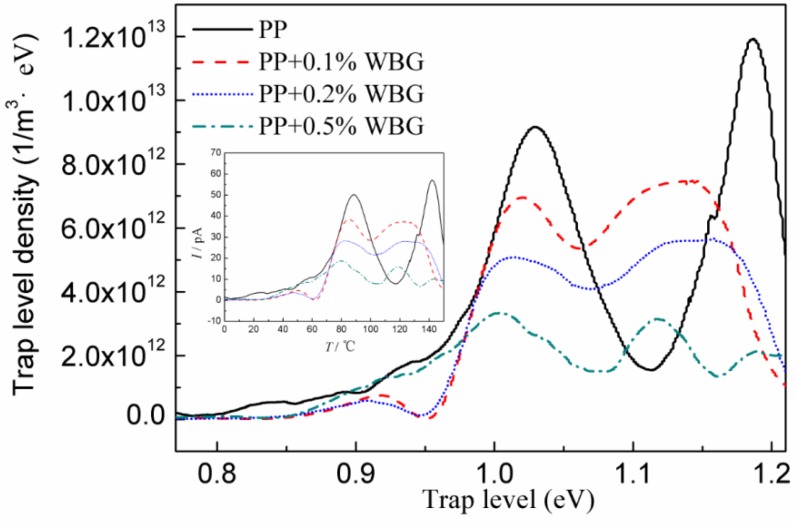
Thermally stimulated depolarization current (TSDC) spectra and the approximated trap depth and density of PP and *β*-PP.

The TSDC spectra and the approximated trap depth and density of PP and *β*-PP are shown in Figure 9. The TSDC spectra present two obvious released current peaks at 85 °C and 140 °C, respectively. Compared with pure PP, the TSDC current peaks of *β*-PP represent lower intensity and temperature. Comparing the DMA spectrum and the TSDC spectrum, it can be found that the loss factors of PP and *β*-PP are consistent with the change of the TSDC value around 85 °C, which decreases with the increase of the nucleating agent loading, and the peak position shifts slightly to a lower temperature. So, the low-temperature TSDC peak around 85 °C more likely comes from the release process of the charge trapped between lamellae due to the relatively low energy required for the relaxation of the polymer chain segment in this region. It can be seen from the DSC melting curve that the initial melting temperature of PP is approximately 140 °C. Observing the loss factor of PP at 140 °C, it can be found that the loss factor of pure PP begins to rise rapidly. In *β*-PP, the same phenomenon occurs at a lower temperature, which is about 10 °C lower than pure PP. The melting initial temperature represents the pre-melting process of the crystal zone, so the peak of the TSDC current at a high temperature around 140 °C comes from the release process of the trapped charge in the crystal region, accompanied by partial melting of the crystal phase. 

In addition to the above two obvious TSDC current peaks, a shallow trap level of about 0.9 eV can be found in the low-temperature range, corresponding to the trap depth illustrated in Figure 7. It can be seen from Figure 7 that the space charge in the *β*-PP is more trapped in these relatively shallow traps, thereby obtaining a greater space charge short circuit decay rate and carrier mobility, while the space charge in PP is more trapped in the deep traps in 1 eV or a deeper trap level. One possible reason is that the density of deep traps in pure PP is significantly higher than that of *β*-PP, resulting in more charge captured by deep traps. The space charge in the polymer is closely related to structural defects [33]. Compared with DSC, DMA and TSDC spectra, there is a clear correlation between them. The *β* nucleating agents reduce structural defects in PP crystal regions and also reduce the number of deep traps in crystal regions. The lower trap depth and lower deep trap concentration may be the reason why the space charge is lower in *β*-PP.

In comparison with Figure 8 and Figure 9, it is also found that the *β*-relaxation process presented in the DMA test has not been measured in the TSDC test because the polarization temperature of the TSDC test (60 °C) is higher than the *β*-relaxation peak temperature. The trap in the amorphous region can no longer stably capture charge carriers at 60 °C temperature, which means the space charge cannot be formed, and the subsequent TSDC measurement of the temperature-increasing process cannot contribute to this part of the trap information. The corresponding *α*-relaxation peak exists at the temperature region higher than 60 °C, hence the charge carriers can be captured by traps in interlamellar and crystal regions to accumulate space charges, which can be tested by TSDC. In addition, the lower temperature region, such as −20 °C, has not been adopted in TSDC tests. This is because the electrode cannot emit notable charges into the sample by Schottky emission, and the charge carrier will transport with very low mobility in this range, resulting in most traps being unfilled with charge so that the TSDC spectrum is invalid.

## 4. Conclusions

The following conclusions are drawn from our experimental research on the structure and properties of polypropylene specifically modified by rare earth nucleating agents: (1) The *β*-nucleating agent, WBG, can effectively induce PP crystallizing in the *β*-crystal form, and 0.2% WBG remarkably increases the relative content of the *β*-crystal to 42.2%; (2) scanning electron microscopy reveals that the *β*-spherulites are characterized by a bunchy shape without a definite boundary between them, and their lamellae are parallel to each other; (3) as the content of *β*-nucleating agents increases, the space charge accumulation in *β*-PP is suppressed, while the decay rate of the space charges increases during short circuit conditions; (4) DMA tests indicate that the *β*-crystal being efficiently induced by WBG can significantly increase and decrease the densities of *β*-relaxation and *α*-relaxation, respectively. The results demonstrate that nucleating agents have changed the defect distribution in PP, resulting in a reduced and increased number of structural defects in the crystal and amorphous regions, respectively. TSDC measurements further verify that the traps in the amorphous region of *β*-PP will not introduce space charges at the working temperature of insulating materials, while the decreased number of traps in the crystal region of *β*-PP can suppress space charge accumulation at ambient temperatures. 

## Figures and Tables

**Table 1:** XRD analysis of PP and *β*-PP.

Samples	Diffraction Peak Intensity				
	*H_α_*_(110)_	*H_α_*_(040)_	*H_α_*_(130)_	*H_β_*_(300)_	*K_β_*, %
PP	35,170	11,764	7011	0	0
PP + 0.1% WBG	36,343	2721	2683	7811	15.8
PP + 0.2% WBG	22,738	4538	4186	24,546	43.8
PP + 0.5% WBG	5963	3645	445	85,655	89.5
